# Sleep-wake circadian rhythm pattern in young adults by actigraphy during social isolation

**DOI:** 10.5935/1984-0063.20220017

**Published:** 2022

**Authors:** Dora Zulema Romero Díaz, Maria Beatriz Duarte Gavião

**Affiliations:** 1 University of Campinas, Piracicaba Dental School, Department of Biosciences -Piracicaba - São Paulo - Brazil.; 2 University of Campinas, Piracicaba Dental School, Department of Health Sciences and Pediatric Dentistry - Piracicaba -São Paulo - Brazil.

**Keywords:** Actigraphy, Circadian rhythm, Sleep, Daylight Cycle, Social isolation

## Abstract

**Study Objectives:**

This study investigated, through wrist actigraphy, the activity-rest pattern, estimate nocturnal sleep parameters, and quantify the exposure of light (daylight and blue light) during social isolation due to COVID-19.

**Methods:**

The participants (n = 19, aged 19 - 33 years-old) wore the actigraph in nondominant wrist for 7 days. Derivation of 25 nocturnal sleep parameters was inferred from PIM mode raw data including sleep, wake, activity, and fragmentation statistics. A hierarchical cluster analysis determined the participants profiles. Mann-Whitney and independent Student t tests, linear stepwise regression and Kendalls test were applied. The significant level was a = 0.05.

**Results:**

Two clusters were formed, normal sleepers (n = 13) and short sleepers (n = 6). The participants of both clusters went to sleep after midnight, spent approximately 1 h of being awake during time in bed, their latency to persistent sleep was normal, though true sleep minutes was less than 7 h, showed a normal sleep efficiency. Daytime activity was moderate, and a circadian rhythm was irregular. The regressions showed that bedtime and nocturnal activity contributed to the variance of daytime activity and the beginning of it (p< 0.001). The midpoint during the time in bed was the most significant predictor for the start of less period activity at night (p< 0.001).

**Conclusions:**

Actigraphy inferred that during social isolation the individuals presented, despite normal sleep latency and efficiency, inconsistent sleep parameters and irregular circadian rhythm. Moreover, decreased exposure to daylight during the morning was observed.

## INTRODUCTION

The pandemic due to Coronavirus disease (Covid-19) is today, in Brazil, a phenomenon of great magnitude and extension, which has caused such great losses in terms of human lives, economy and quality of life of individuals.

The 17th of March 2020, the São Paulo Government implemented measures to prevent risks of direct transmission of COVID-19. The Brazilian population has been subjected to a substantial period of social isolation with a restriction of movements, cancellation of all events promoted by the Governments that generate crowds of people, such as events sporting, artistic, cultural, political, scientific, and commercial. Many people have been in home confinement situations; non-essential services adopted a non-face-to-face regime (e.g., education in every level), and were authorized to operate the services of urgent needs of the community (health, food, and security). These changes in lifestyle have negative consequences for well-being, which impact sleep quality that can be related to changes in the sleep/wake cycle and circadian rhythms^1,2^. Circadian rhythms are 24 h daily cycles that can be entrained or phase- shifted not only by our internal clock but also by external factors such as daily schedules, social rhythms, and daylight exposition^3^. This is important because activity and behavior during wakefulness can infuence the duration and quality of sleep and, conversely, the duration and quality of sleep can affect daytime function^4^.

Previous studies have been presented using online questionnaires, as the lifestyle changes during social isolation^5,6^, not allowing the clinical evaluation of research participants. Nevertheless, studies are required to show objective data on the rhythmicity of the circadian cycle and sleep in healthy individuals during that time. In this sense, this study hypothesizes that social isolation can alter the sleep–wake pattern, generating irregular and less robust activity–rest patterns. Furthermore, during this period the daily exposure daylight may have decreased because people stayed at home, which may have led to increased exposure to blue light. Thus, this study aimed to investigate, through wrist actigraphy, the activity–rest pattern, estimate nocturnal sleep parameters, and quantify the exposure of light (daylight and blue light) in healthy individuals who were in social isolation during the COVID-19 pandemic.

## MATERIAL AND METHODS

This study was approved by the Ethics Committee of of Piracicaba Dental School, University of Campinas (FOP-UNICAMP) (ethical approval CAAE 95764718.6.0000.5418). The participants provided their written informed consent to participate.

### Participants

The base population of this study were well-known young adults, specifically students from the high-schools and universities of Piracicaba, S P, Brazil. First, they were considered as potential participants and then were invited to participate via WhatsApp, composing a convenience sample. The inclusion criteria were age 18 to 35 years, body mass index (BMI) of 18.5–24.9 kg/m^2^, to sleep alone (to ensure that the activity records during bedtime were owned by the participant, avoiding false positives generated by the movements of a partner), to be in social isolation and living in Piracicaba city, São Paulo state, Brazil. Individuals who were self-reporting sleep disorders and respiratory diseases by a standardized questionnaire, for example, obstructive sleep apnea and asthma, and neurological disorders were excluded. Demographic data, such as age, sex, marital status, and education were obtained through questionnaires. To calculate the BMI, self-reported height and weight were obtained from which BMI was calculated as weight in kilograms (kg) divided by height in square meters (m^2^).

### Study design

In the framework of chronobiological study designs, longitudinal sampling corresponds to obtaining data on the same individual as a function of time^7^. In this study, 24 h periods were recorded for seven consecutive days; however, this study was cross-sectional and descriptive-observational.

The data were collected from March 16^th^ to June 8^th^, 2020. The degree of social isolation began from March 17^th^ in the State of São Paulo, Brazil, when the acquisition of actigraphy data began. Schools were closed, the home-office was encouraged and activities with many people were restricted. However, the measures adopted of social isolation were not so strict, which gave people relative freedom, i.e., they could decide to stay at home and go out only if necessary or go out to walk/run. Initially, the participants should receive the actigraph in the lab of the Dental School, but due to the closure of educational institutions, the device has just been delivered and removed at the participants’ homes.

**Figure 1:**
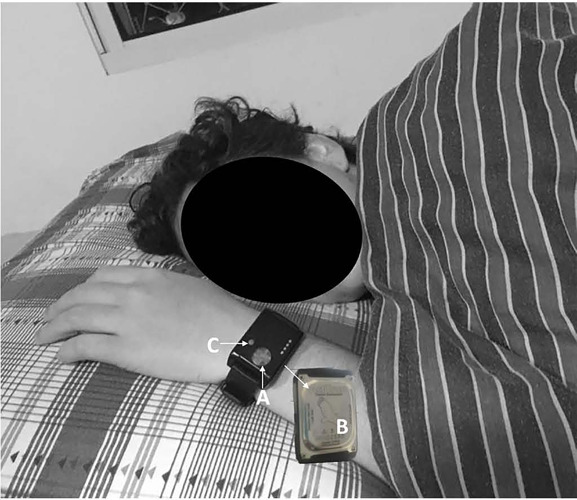
The circadian monitoring device, composed of an accelerometer (internal, not shown in the figure), luxometer (A), a temperature sensor (B) and event-marker button (C).

The actigraph used was the ActTrust® (model AT0503 Condor Instruments, Brazil) applied to evaluate the circadian rhythms and sleep parameters of the selected sample. This device was previously validated by^8^, showing excellent sensitivity (95.69%), good accuracy (80.24%), predictive value for the sleep of 81.52% and predictive value for wakefulness of 68.93% in individuals with sleep-disordered breathing. All participants were instructed to use the device on the wrist of the non-dominant hand for 7 days (Fig. 1), starting and ending on Monday at midday (including weekends) and fill in the sleep diary simultaneously; and to maintain their normal lifestyle during the week of data collection.

Standardized procedures were performed on the device configuration (ActStudio 1.013®, Condor Instruments, Brazil) before handing it over to the participant, as follows:

- The device was programmed to record activity counts in the proportional integral mode (PIM), every 30 s, and added to generate a 1 min epoch; similarly, light was recorded every 30 s.
- The calculations of day length were based on the times of sunrise and sunset (corresponding with the start of the light and dark phase of the 24 h period, respectively) computed based on the local latitude, longitude, date, and geopolitical time zone.
- The information stored in the actigraph was transferred via a USB adapter (ActDock®, Condor Instr uments, Brazil) to a computer using the software provided by the manufacturer (ActStudio 1.013®, Condor Instruments, Brazil). All configurated were made on the same computer.
- The examiner (DZRD) was previously trained to handle actigraphy related to data acquisition and analysis. Five participants, aged 24.6 ± 4.16 years, properly instructed, used the device for four consecutive days. After 12 days, the data were acquired again in the same way allowing the determination of intra-examiner reliability using the intraclass correlation coefficient, which was 97-99, showing “good” reliability, according to Koo and Li^9^. These participants were not included in the final study.

### Measures

The sleep parameters were calculated based on the Cole-Kripke algorithm^10^ and are included in four statistics, namely, sleep, wake, activity, and fragmentation, providing a detailed analysis method to infer these parameters objectively, allowing a description of events during the periods^11^. The derivation of each sleep parameter uses only the sleep scores. The complete definition of each parameter can be seen in the supplementary materials, as well as the normal values and ranges, so the studied variables were listed below:

*A) Sleep statistics*
  i. Time in bed (TIB)
  ii. Sleep period (SLP)
  iii. Sleep minutes during TIB (SMIN)
  iv. True sleep minutes (TSMIN)
  v. Sleep onset latency (SOL)
  vi. Latency to persistent sleep (LPS)
  vii. Percent sleep (PSLP)
  viii Sleep efficiency (SE)
  ix. Sleep episodes (SEP)
  x. Mean sleep episode (MSEP)
  xi. Long sleep episodes (LSEP)
  xii. Longest sleep episode (LGSEP)
*B) Wake statistics*
  i. Wake minutes during TIB (WMIN)
  ii. Wake after sleep onset (WASO)
  iii. Number of awakenings during TIB (NA)
  iv. Mean wake episode (MWEP)
  v. Long wake episodes (LWEP)
  vi. Longest wake episode (LGWEP)
*C) Activity statistics*
  i. Mean activity during TIB (AMEAN)
  ii. Activity standard deviation during TIB (ASD)
  iii. Activity index (ACTX)
*D) Fragmentation statistics*
  i. Sleep fragmentation index (SFX) ii. Brief wake ratio (BWR)
*Circadian rhythm non-parametric variables*
  i. Activity counts for the most active 10 h period (M10) and Onset-M10
  ii. Activity counts for the least active 5 h period (L5) and Onset-L5
  iii. Inter-daily stability (IS)
  iv. Intra-daily variability (IV)
  v. Relative amplitude (RA)
  vi. Circadian function index (CFI)
*Complementary measures*
  i. Chronotype by SLP and TIB
  ii. Exposure to daylight and blue light

### Statistical analyses

For data statistical analyses, the Statistical Package for Social Sciences, version (SPSS® Statistics Inc., Chicago, USA) was used. Descriptive statistics were performed for all variables and sample characterization expressed as mean, standard deviation, median, and quartile amplitude.

To study the participants’ profiles, a cluster analysis was performed, which aims to organize a set of cases into homogeneous groups, in such a way that the individuals belonging to a group are similar to each other and different from the rest^12^. The hierarchical cluster analysis was performed using the farthest neighbor method for calculating distances between clusters and obtain the dendrogram. The sleep parameters that contributed to the formation of clusters can be seen in Supplementary Table 1^12^. After analyzing the plot dendrogram, it was decided to inform *a priori* the number of clusters to be performed for identifying clusters of participants with similar sleep parameters. Thus, two clusters were chosen (clusters 1 and 2). The differences between clusters were assessed by the Mann–Whitney test for clustering validation.

**Table 1:** Characteristics of the study population.

Age (years)^a^	25.4 ± 4.3
Gender (N, %)	n = 19
Male	9 (47%)
Female	10 (53%)
BMI (kg/m^2^)^a^	21.8 ± 3.07
Education (N, %)	
High school graduate	2 (11%)
College	8 (42%)
Postgraduate	9 (47%)
School type (N)	
Private	5 (26%)
Public	14 (74%)

^a^No sex difference.

Data distribution was assessed using the Shapiro–Wilk test and the quartile–quartile plot (QQ-plot) graphs, and the homogeneity Levene test. The sleep parameters and circadian rhythms (M10, L5, and RA) show data not normally distributed, whereas the circadian rhythms (IS, IV, and CFI) were normally distributed. To compare the differences between clusters and differences between weekdays and weekends, the Mann–Whitney test was applied. For the variables I V, IS, and CFI the differences between clusters, the independent Student t-test was applied.

Exploratory analyses were conducted to investigate potential relationships between circadian rhythm variables (M10 and L5) and sleep parameters predictors (i.e., Onset SLP and ASD, etc.). The relationships were tested using the linear stepwise regression model. The blue light and sunlight intensity were correlated with Onset SLP and Onset L5, using Kendall’s test.

The level of significance was defined as alpha equal to 0.05.

## RESULTS

### Sample

The characteristics of the study population are shown in Table 1. Initially, 21 individuals were assessed for eligibility. However, two were excluded, because one did not sign the informed consent and the other returned to work during the week of participation. Finally, nineteen (male n = 9; female n = 10) adults were included in the final convenience sample. The participants lived in the same geographic area (urban region of residence).

### Sleep patterns

Table 2 shows the values of sleep patterns. The cluster analysis generated two groups varying significantly according to the sleep parameters. Using the mean and median of the TIB and SLP, the clusters were being nominated as “normal sleepers” (cluster 1, n = 13, SLP > 7h) and “short sleepers” (cluster 2, n = 6, SLP < 5.5h). Despite the late sleep times, normal sleepers were characterized by better sleep parameters, such as a normal sleep duration with almost two more hours than short sleepers. The participants of both clusters went to sleep after midnight: normal sleepers at 01h33 ± 10.33 and short sleepers at 03h04 ± 7.03. Other parameters of sleep statistics, such as TSMIN, PSLP, SE, MSEP, and LGSEP were significantly higher also for normal sleepers than for short sleepers, which indicates better sleep. However, the parameter TSMIN for normal sleepers showed values slightly below normal, and for short sleepers, the values were even lower. The SOL and SE values were similar for both clusters but were within the normal range.

**Table 2:** Comparison of the sleep parameters by clusters.

Sleep parameters	Normal sleepers (n = 13)		Short sleepers (n = 6)		U (*p*)
	Me ± SD	Md (25 ^th^ - 75 ^th^)	Me ± SD	Md (25 ^th^ - 75 ^th^)	
A. Sleep statistics					
Bedtime (hh:mm)	01:33 ± 10.56	02:40 (00:30 – 03:33)	03:04 ± 7.03	02:45 (01:19 – 04:40)	1800.00 (0.823)
Get up time (hh:mm)	08:59 ± 1.88	09:00 (08:05 – 10:30)	09:09 ± 2.09	09:00 (08:14 – 10:39)	1733.00 (0.578)
TIB (h)	8.19 ± 1.11	8.18 (7.31 – 8.50)	6.46 ± 1.32	7.40 (6.42 – 8.29)	702.00 (<0.001)^a^
SLP (h)	7.18 ± 1.10	7.19 (6.44 –7.48)	5.40 ± 1.41	6.38 (5.24 – 7.36)	665.00 (<0.001)^a^
Onset SLP (hh:mm)	02:11 ± 9.33	02:18 (01:51 – 03:52)	04:27 ± 4.83	03:09 (02:54 – 04:48)	1613.00 (0.249)
Offset SLP (hh:mm)	08:42 ± 1.92	08:45 (08:04 – 10:10)	08:56 ± 2.25	08:55 (07:44 – 10:32)	1693.50 (0.452)
SMIN (h)	7.14 ± 1.10	7.15 (6.42 – 7.51)	5.33 ± 1.27	5.46 (5.21 – 7.23)	494.50 (<0.001)^a^
TSMIN (h)	6.48 ± 1.02	6.52 (6.16 – 7.18)	5.03 ± 1.30	5.07 (4.38 – 6.56)	498.00 (<0.001)^a^
SOL (min)	14.33 ± 16.23	9.50 (5.75 – 16.25)	18.29 ± 25.02	12.00 (5.50 – 18.50)	1665.00 (0.370)
LPS (min)	43.41 ± 22.76	34.00 (27.00 – 56.25)	53.00 ± 41.13	38.00 (29.50 – 62.50)	1645.00 (0.320)
PSLP (%)	82.58 ± 6.50	83.04 (78.15 – 88.20)	74.95 ± 12.84	78.28 (70.24 – 83.23)	1138.50 (<0.001)^a^
SE (%)	93.26 ± 4.80	94.34 (89.72 – 96.97)	89.43 ± 7.22	91.01 (87.75 – 94.23)	1241.00 (0.003)^a^
SEP (#)	10.96 ± 5.40	9.50 (7.00 – 15.00)	12.02 ± 5.05	11.00 (9.50 – 14.00)	1533.50 (0.121)
MSEP (min)	50.30 ± 27.54	43.02 (28.80 – 65.26)	35.31 ± 30.25	30.17 (19.87 – 39.35)	1059.00 (<0.001)^a^
LSEP (#)	8.89 ± 3.93	8.00 (6.00 – 11.00)	9.56 ± 3.59	9.00 (7.00 – 12.00)	1589.50 (0.203)
LGSEP (min)	152.08 ± 80.98	124.00 (94.75 – 84.25)	108.24 ± 72.26	89.00 (65.50 – 124.00)	1058.50 (<0.001)^a^
B. Wake statistics					
WMIN (min)	60.29 ± 31.36	58.50 (36.00 – 75.00)	73.30 ± 54.24	57.00 (40.00 – 89.00)	1755.50 (0.657)
WASO (min)	29.52 ± 22.15	23.00 (12.75 – 45.25)	36.07 ± 28.33	30.00 (18.50 – 39.00)	1566.50 (0.167)
NA (#)	8.17 ± 5.15	7.00 (4.00 – 11.00)	8.85 ± 5.01	8.00 (5.50 – 10.50)	1645.00 (0.320)
MWEP (min)	6.77 ± 5.32	4.85 (3.83 – 7.53)	6.53 ± 3.81	5.36 (4.09 – 7.51)	1722.50 (0.543)
LWEP (#)	3.77 ± 2.11	3.50 (2.00 – 5.00)	4.93 ± 2.94	4.00 (3.00 – 6.50)	1443.00 (0.043)^a^
LGWEP (min)	23.21 ± 19.91	15.00 (11.00 – 30.25)	27.05 ± 36.07	14.00 (11.00 – 22.50)	1786.00 (0.769)
C. Activity statistics					
AMEAN (counts)	220.02 ± 117.41	198.89 (149.31 – 250.26)	294.72 ± 180.17	245.30 (196.20 – 337.47)	20.00 (0.096)
ASD (counts)	798.88 ± 333.98	751.26 (595.94 - 927.05)	962.31 ± 363.23	925.15 (672.10 – 1113.66)	20.00 (0.096)
ACTX (%)	23.78 ± 7.25	22.93 (18.44 – 29.28)	25.15 ± 9.84	24.73 (20.56 – 28.75)	1390.50 (0.599)
D. Fragmentation statistics					
SFX (%)	1.91 ± 1.23	1.50 (0.94 – 2.69)	2.77 ± 1.65	2.35 (1.79 – 3.39)	1237.50 (0.003)a
BWR (A1′/NA)	0.11 ± 0.10	0.10 (0.00 – 0.18)	0.12 ± 0.09	0.11 (0.04 – 0.17)	1726.50 (0.551)
Complimentary measures					
Chronotype					
Midpoint of the TIB (hh:mm)	05:08 ± 1.50	04:47 (04:16 – 05:40)	05:52 ± 1.83	05:11 (04:28 – 07:26)	1338.50 (0.012)a
Midpoint of the SLP (hh:mm)	05:12 ± 1.66	04:58 (04:03 – 05:58)	06:07 ± 2.04	05:55 (04:40 – 07:59)	1252.00 (0.003)a

Data are presented as mean and standard deviation (Me ± SD) and median and quartile (25^th^ percentile – 75^th^ percentile) TIB = time in bed; SLP = sleep period; NA = number of awakening; A1′ = awakenings lasting only 1 min; ^a^Significantly different (p < 0.05) between clusters in the Mann–Whitney test.

LWEP and SFX all parameters were significantly lower for normal sleepers.

Nevertheless, the similar values of NA between clusters were above the normal range.

Although the other parameters were similar for both clusters, in the pre-selection, they were considered different as much as possible.

Differences between weekdays and weekend was only for SLP, since short sleepers had lower values, suggesting that they slept one hour and thirty minutes less during the weekend when compared with the normal sleepers (p < 0.005) (Supplementary Table S2 for differences between weekdays and weekends data).

### Circadian rhythm variables derived from non-parametric approaches

The circadian rhythms variables (Table 3) derived from non-parametric approaches showed that the two clusters presented moderate patterns of activity for M10 indicating active waking periods. Although Onset M10 started late in both clusters, normal sleepers started an hour and a half before than did short sleepers. L5 values were low, but significantly lower for normal sleepers than for short sleepers, indicating that the two clusters had less restful sleep (nocturnal activity). This finding is in accordance with the activity statistics in Table 2. In addition, L5 started very late for both clusters, accordingly to Onset-L5 time, but significantly earlier for normal sleepers. IV values suggested the occurrence of nocturnal awakenings in both clusters, with no significant differences; this finding is in accordance with the wake statistics in Table 2. The repetitiveness of the rhythm across consecutive days showed low synchronization, as IS mean and median values were below 0.5. There was a subtle but significant difference in RA between clusters, suggesting that the short sleepers showed more irregular circadian rhythm when compared with normal sleepers. The CFI in both clusters showed low values, indicating low circadian rhythmicity.

**Table 3:** Circadian rhythms variables derived from non-parametric approaches by cluster.

Circadian rhythms variables (PIM^†^)	Normal sleepers (n = 13)		Short sleepers (n = 6)		
	Me ± SD	Md (25^th^ – 75^th^)	Me ± SD	Md (25^th^ – 75^th^)	U (*p*)^a^/ t (*p*)^b^
M10 (count)	3463.42 ± 1863.35	3129.22 (2397.77 – 3816.21)	3405.40 ± 2250.41	2658.42 (2047.82 – 4133.92)	1994.00 (0.306)^a^
Onset M10 (hh:mm)^♦^	09:41 ± 3.17	09:43 (08:25 – 11:57)	10:00 ± 4.31	11:18 (09:47 – 12:47)	1807.50 (0.067)^a^
L5 (count)	312.61 ± 589.63	107.06 (71.88 – 167.58)	380.40 ± 613.50	159.34 (109.64 – 292.13)	1510.00 (0.002)^a^
Onset L5 (hh:mm)^♦^	03:43 ± 5.09	01:39 (00:43 – 03:38)	04:54 ± 4.91	03:18 (02:09 – 05:09)	1449.00 (<0.001)^a^
IS*** (A.U. 0–1)	0.42 ± 0.09	0.40 (0.34 – 0.47)	0.33 ± 0.11	0.32 (0.23 – 0.49)	−1.89 (0.075)^b^
IV** (A.U. 0–2)	0.77 ± 0.19	0.74 (0.65 – 0.92)	0.79 ± 0.16	0.74 (0.68 – 0.87)	0.20 (0.842)^b^
RA* (A.U. 0–1)	0.84 ± 0.23	0.93 (0.87 – 0.96)	0.80 ± 0.21	0.90 (0.77 – 0.93)	1677.00 (0.017)^a^
CFI**** (A.U. 0–1)	0.44 ± 0.08	0.44 (0.39 – 0.53)	0.43 ± 0.08	0.44 (0.34 – 0.49)	−0.49 (0.627)^b^

Data are presented as mean and standard deviation (Me ± SD) and median and and median and quartile (25^th^ percentile - 75^th^ percentile); ^†^Proportional Integration Mode; *Relative amplitude; **Intra-daily variability; ***Inter-daily stability; ****Circadian function index; ^♦^Indicates the decimal hour when the activity count begin; A.U. = arbitrary units; ^a^p < 0.05 in the Mann-Whitney test; ^b^p < 0.05 in the independent Student t-test.

**Figure 2:**
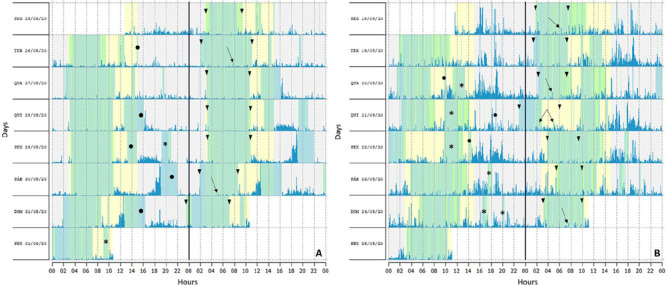
The actograms of the wrist actigraphy. A degree of variability was evidenced during the 7 days. (A) Actogram of one participant of the normal sleeper cluster. The almost vertical arrangement of the arrows indicates a tendency toward regularity at bedtime. (B) Actogram of one participant of the short sleeper cluster. The vertical alignment of the ▼ shows an irregular pattern, indicating that sleep periods did not happen at the same time during night.* moments when the actigraph was off the wrist; ▼ ▼ time in bed; ● moments of silence awake in bed.

Figure 2 (A) shows the actogram data from a participant in the normal sleeper cluster and (B) from a participant in the short sleeper cluster, demonstrating minute-by-minute wrist movement values (activity counts) over 7 days. A double-plotted graph enables a clearer observation of the data.

Table 4 shows the significant models of stepwise multivariate regression considering the circadian rhythm (M10 and L5) as dependent variables and the respective sleep parameters as the predictors. M10 could be explained by 21% of the sleep parameters. ASD was also a significant predictor for M10. AMEAN per night infuenced significantly L5, explaining 21% of the variability. Sleep parameters could predict the Onset L5 very well, explained 81% of the variability. The parameters were midpoint TIB, LGSEP, and TSMIN, with the latter two negatively infuencing the models.

**Table 4:** Model comparison and results of the linear stepwise regression models predicting circadian rhythm variables.

		Coefficient statistics			
Dependent variable	Predictor	Coeff.^†^ (SE)	β^‡^	CI	t (p)
*M10-PIM: [F(1,17) = 4.78; p = 0.043; R^2^ =0.46]*					
	ASD	4.88 (2.23)	0.46	[0.17 – 9.59]	2.18 (0.043)
*Onset-M10-PIM: [F(2;16) = 10.10;p = 0.001; R^2^ = 0.55]*					
	Bedtime	−0.08(0.02)	−0.49	[−0.42 - 0.02]	−2.85 (0.012)
	WASO	0.03(0.01)	0.45	[0.006 - 0.58]	2.63 (0.018)
L5—PIM: [F(1,17) = 5.85; p = 0.027; R2 = 0.50]					
	AMED	0.82 (0.343)	0.50	[0.10 - 1.55]	2.42 (0.027)
*Onset-L5—PIM: [F(2, 16) = 34.12; p < 0.001; R^2^ = 0.81]*					
	Midpoint of the TIB	0.67(0.09)	0.76	[0.46 - −0.88]	6.89 (<0.001)
	LGSEP	−0.008(0.003)	−0.35	[−0.01 - −0.003]	−3.15 (0.006)

Equation: [(degrees of freedom, regression – residual)] F-value; p-value; R^2^]; ^†^unstandardized regression coefficient (SE: standard error); ^‡^standardized regression coefficient; CI = 95% confidence intervals obtained using stepwise method. ^a^Spearman′s correlation (p-value) between independent variables. Limits of variable: collinearity statistics [tolerance: 0.17–1.00; VIF: 1.00–5.74]; residual statistics [std. predictive: −0.98, −3.47; std. residual: −2.06, 2.13].

The other sleep parameters did not meet the presuppositions for linear regression.

### Exposure to light during the light–dark cycle (i.e., 24 h period)

**Figure 3:**
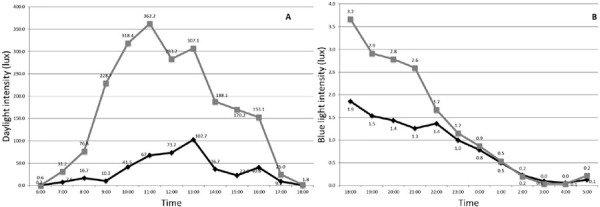
Intensity of light during the 24-hour period. A) The exposure to daylight. B) The trajectory of the intensity of blue light. The black line represents normal sleepers and gray line represents the short sleepers.

Figure 3 shows the mean light level. Figure 3A shows the exposure to daylight from 06h00 to 18h00, whereas Figure 3B shows the trajectory of the intensity of blue light from 18h00 in the afternoon to 05h00 in the morning. Both light levels were of the light–dark cycle. The normal sleepers (gray line) showed significantly higher exposure to daylight (U = 37.00; p = 0.015) than short sleepers (black line). However, there were no significant differences (U = 61.00; p = 0.525) in evening blue light exposure between clusters. The Supplementary Table S3 shows all values of daylight and blue light exposition, respectively. There was no significant correlation between daylight and blue light with onset L5 or onset SLP (all rs < 0.36, p > 0.12) (Supplementary Table S4).

## DISCUSSION

As the COVID-19 pandemic changed lifestyle during the first wave worldwide, mainly in terms of social isolation, this study aimed to assess sleep parameters in healthy individuals during this period and compare them with parameters established in the literature.

Bedtime can be considered delayed in the present study, probably due to isolation during the COVID-19 pandemic. This is an important observation, since later sleep timing has been associated with poor health results^13,14^ and it can be altered by social isolation, as found in previous studies in which participants showed significantly delayed in time to go to bed during quarantine compared to pre-quarantine time^15,16^. The SLP in the short sleepers was much less than the recommended time of at least 7 h stated by the American Academy of Sleep Medicine and the Sleep Research Society, as well as the TSMIN, despite TIB has been about 7 h in this cluster. On the other hand, normal sleepers had SLP more than 7 h, but TSMIN was below 7 h on average. The low values for TSMIN can be related to awakenings episodes, as observed in pre-pandemic period by Cellini et al.^17^, using actigraphy (Actiwatch-64). New sleep behaviors can be assumed during social isolation due to changes in daytime and night-time activities and routines can infuence the parameters mentioned above^18,19^. An interesting observation is the similarity between median values of Onset SLP (calculated) and the values of Onset L5 (the device’s algorithm) mainly in the short sleepers. This finding shows the need for further studies that implement a methodological analysis for the derivation of the sleep parameters presented in this study to test whether the Onset SLP and the Onset L5 match.

Get up was nearly 9 a.m., without difference between weekdays and weekend. Recent studies have found that during social isolation, the sleep-wake time difference between weekdays and weekend days decreased^16,20^ due to a delay in mid-sleep on workdays^21^. One possibility is that social isolation increased fexibility regarding social schedules, determining the postponed time of awakening on workdays^20^.

The SOL showed normal values in both clusters, despite the late bedtime, and was similar with other studies before Covid-19 pandemic^17,22,23,24^. Conversely, Korman et al.^20^, observed a delay in sleep onset during social isolation that did not affected the sleep duration, due to the postponed wake-up time. The respective differences can be attributed to different measurements, since the study of Korman et al.^20^, used questionnaires in a large sample, whereas the others used actigraphy.

Moreover, the sleep parameters, WASO and SE, presented also normal values, suggesting that, although participants slept late and presented light activity during TIB, sleep quality was adequate, that is, greater than 90% in both clusters. These findings agree with previous studies using other devices^22,24,25,26^ but are in contrast with Cellini et al.^27^ and Haghayegh et al.^28^, who showed that participants spent adequate TIB, but the sleep quality was not good enough, mainly because of a high amount of WASO, differing from the participants in the present study. Some studies during social isolation due to Covid19 pandemic agreed with Cellini et al.^27^, showing a decrease in sleep quality in this time^15,21^. On the other hand, many people experienced increased fexibility regarding social schedules, leading to improve individual sleep– wake timing and overall more sleep^16,21^, that can be occurred in part with the participants of the present study.

The chronotype was estimated by midpoint of the TIB and SLP, as done also by Wright et al.^16^; circadian phase or questionnaire to estimate chronotype were not considered. There was significantly difference between clusters, with a later chronotype for the short sleepers, although no differences were found between clusters for sleep-wake-up time, considering TIB and SLP. This was an unexpected result, as late chronotypes prefer to wake up later in the morning and sleep later at night^11^, but the condition of social isolation during the pandemic could be an infuencing factor. Moreover, variations in chronotype can be associated with variations in the timing of numerous physiological and behavioral variables^16,29^.

The activity pattern for M10 in this study did not differ between clusters, suggesting that the participants perform a moderate activity in the wake period during daytime, as seen also by Forner-Cordero et al.^30^. According to Pépin et al.^31^, the activity pattern did not appear to have a significant clinical impact on people’s activity compared to the pre-pandemic period. M10 was in line with L5 values, indicating that participants presented slight movement intensity during the time of less activity that usually occurs during the sleep period^32^, agreeing with previous studies^23,30^. Differences in circadian rhythm between clusters can be explained because during the social isolation the first one had a little bit to exposure to light during the early hours of the morning than the second one, which can lead to the delay of the biological clock, since sunlight has been considered as an important zeitgeberts^33,34^.

The stepwise regression models showed that both sleep parameters, M10 and L5, explained a significant amount of the variance in the level of day/night activity. Of the various factors that may infuence M10, ASD contributed significantly to the variation of daytime activity, demonstrating that activity during the sleep period may have had the effect of decreasing the amount of daytime activity. This is an interesting result since activity at night can stabilize day-to-day habits, thus creating a more regular circadian rhythm^35^. However, during the COVID-19 pandemic, the participants were kept in their homes, probably reducing the physical activity.

A relevant finding of the study was that the bedtime was a significant and negative contributor to the beginning of daytime activity because a late bedtime overlaps sleep in the middle of the morning, consequently it will lead to a few hours of daytime activity. The lack of activity significantly increases the fragmentation of the activity–rest rhythm^35^, which was observed in the present sample. WASO was other significant contributor, suggesting that fragmented nights by nocturnal activity can also contribute to Onset M10 delay. Moreover, AMEAN significantly infuenced L5 due to nighttime activity during the least active period of the light–dark cycle. The preference time to sleep and wake up (Midpoint TIB) was the most significant predictor for the variation of the Onset-L5, because the participants of this study preferred to go to sleep late and wake up later. This sleep behavior produces a delay at the beginning of M10 during the light–dark cycle, and consequently, it makes L5 start later.

In Cluster 1, the daylight intensity increases at 09h00 exceeding the 200-lux threshold, which can indicate exposure to daylight at that time. However, in the Cluster 2, the exposure to daylight remains low, suggesting that they have been indoor for a longer time. According to Korman et al.^20^, social restrictions lead to robust shifts in daily behavior and in exposure to daylight that can explain the present findings. Moreover, participants could be exposed to less daylight during social isolation, due to the characteristics of the house, such as small windows and no outside area^19^. When analyzing the actograms, a degree of variability was evidenced during the 7 days (Figure 2), but a tendency toward regularity at bedtime for normal sleepers was observed, whereas for short sleepers the sleep periods were irregular. In addition, the activity cycle showed out of sync with the light–dark cycle. This is according to a previous study^33^, that reported that the short sleepers who frequently change their sleep timing and consequently their pattern of light–dark exposure showed misalignment between the circadian system and the sleep / wake cycle. In both clusters, the activity cycle is not synchronized to the light–dark cycle, in a 24 h period, because wake up happens in mid-morning ranging from 09h00 to 10h00. The interesting finding of this work is that during social isolation, the wide variation in sleep parameters was characterized as irregular and free-running, and sleep–wake rhythm out of sync.

The intensity of the blue light at 22h30 of the night was few, 1.7 lux^36^, which is possibly the result of the ambient light at home. The blue light intensity decreases at midnight, and both clusters showed low values at 03h00 of the night, indicating darkness during SLP. No significant correlation was observed between low evening blue light levels before Onset L5 or Onset SLP. One hypothesis to explain this fact is the light sensor was outside the emission ratio of the blue light source and that, sequentially, can explain the low levels of light registered. This finding contrasts with the related literature^37,38^ in which chronic exposure to low-intensity blue light directly before bedtime may have serious implications on the circadian phase, resulting in lower subjectively perceived sleep quality. In fact, the infuence of blue light on sleep parameters has been a controversial issue, due to the different methodologies including self-reported or objective assessments. In this context, the findings of the present study are in line with others that evaluated objectively sleep parameters related to blue light exposure. In randomized controlled trials in individuals having insomnia, the use of blue light blocking and behavioral therapy improved self-reported measures of sleep quality, but there was no improvement in total sleep time using actigraphy^38^. Thus, it is possible to infer that blue light did not affect the sleep parameters measured objectively, such as the actigraphy, but other clinical trials must be addressed incorporating other measurements, such as melatonin and polysomnography^39^ to allow a more precise diagnosis.

The findings should be interpreted considering the limitations of the study that include (i) the lack of PSG data to validate findings (the gold standard of sleep assessment). However, the adopted methodology provided a clear and detailed protocol for scoring sleep. Moreover, Rodrigues and Eckelli^8^ observed that the ActTrust® actigraph presented excellent sensitivity and good accuracy. Another limitation is that (ii) data acquisition was in a small convenience sample of healthy adults of different ages only during the period of social isolation, determining that the results cannot be extrapolated to other populations and requiring new assessment of the participants in the post-pandemic period. Further, (iii) the fact that most studies use methodologies and devices different from the present study determine that the respective comparisons should be interpreted with caution. Moreover, (iv) no measures of daily sleepiness and nap were collected, as well as data prior to the period of social isolation; therefore, any strong assumptions about sleep disorders cannot be made. Finally, (v) the cross-sectional design of the study does not allow for the determination of a causal effect of circadian rhythm on sleep parameters.

Despite the aforementioned limitations, it is important to guide individuals in general to maintain a more consistent sleep schedule, with regular sleep time in an adequate and specific environment, use electronic equipment sparingly at appropriate times, avoiding stimulating drinks before bedtime and avoiding light during sleep. These cares and/or habits can contribute to mitigate the sleep disturbances reported in this study.

In conclusion, actigraphy inferred that during social isolation the individuals presented, despite normal sleep latency and efficiency, inconsistent sleep parameters and irregular circadian rhythm. Moreover, decreased exposure to daylight during the morning was observed.

## SUPPLEMENTARY MATERIAL

Sleep Parameters

A) Sleep statistics: refers to the moments of asleep the participant had during time in bed.
  i. Time in bed (TIB) is defined as the time that the participant lies in bed after turning off the lights until the time to physically get out of bed.
  ii. Sleep period (SLP): interval from sleep onset to sleep offset (O–O interval).
  iii. Sleep minutes during TIB (SMIN): the total number of minutes during TIB.
  iv. True sleep minutes (TSMIN): the total number of minutes during SLP. The recommended normal for adults is 7–9 h of sleep per night^1^.
  v. Sleep onset latency (SOL): the number of minutes between lying down in bed and actually falling asleep. The normal limit for adults is less than 20 min.
  vi. Latency to persistent sleep (LPS): the start of persistent sleep at least 20 min.
  vii. Percent sleep (PSLP): the minutes of asleep during TIB; the normal for adults is ≥80%.
  viii. Sleep efficiency (SE): the percentage of time spent asleep during the sleep period; the normal limit for adults is ≥80%.
  ix. Sleep episodes (SEP): the count of instances when the participant was asleep for one or more minutes.
  x. Mean sleep episode (MSEP): the average number of minutes the participant was asleep per sleep episode.
  xi. Long sleep episodes (LSEP): the total number of instances when the participant was asleep for at least 5 min during TIB (considered long if it lasts at least 5 min).
  xii. Longest sleep episode (LGSEP): the duration (in minutes) of the longest sleep episode during TIB.
B) Wake statistics: refers to the moments of awake the participant had during time in bed (the minute-by-minute wrist movement values).
  i. Wake minutes during time in bed (WMIN).
  ii. Wake after sleep onset (WASO): the number of minutes that the participant was awake between sleep onset and sleep offset, considering only the TSMIN; the normal value in adults is <10% of total sleep minutes.
  iii. The number of awakenings during TIB (NA) for one or more minutes; normal values in adults range from 2 to 6 awakenings per night.
  iv. Mean wake episode (MWEP): awakening minutes during time in bed.
  v. Long wake episodes (LWEP): awakening episode for at least 5 min.
  vi. Longest wake episode (LGWEP): the duration of the longest wake episode during time in bed (min).
C) Activity statistics: refers to the activity the participant had during time in bed.
  i. Mean activity during TIB (AMEAN): frequency of wrist movement (PIM) per minute during time in bed.
  ii. Activity standard deviation during TIB (ASD): the variability in the activity score.
  iii. Activity index (ACTX): minutes during TIB where the activity score was greater than zero (0).
D) Fragmentation statistics: are indicators of restlessness or nocturnal movement during time in bed.
  i. Sleep fragmentation index (SFX): the ratio of the NA to the total sleep time in minutes.
  ii. Brief wake ratio (BWR): the ratio of the NA lasting only 1 min to the total NA during TIB.

Circadian rhythm non-parametric variables

Non-parametric analysis was used to calculate the following phase markers^2,3,4,5^.

i. Activity counts for the most active 10 h period (M10) and start of M10 (Onset-M10): refects how active the wake periods are.
ii. Activity counts for the least active 5 h period (L5) and start of L5 (Onset-L5): activity levels during the night. Thus, the patterns of activity can be classified into intensity levels^6^ as follows: light ≤ 1951 counts/min, moderate = 1952–5724 counts/min, hard = 5725–9498 counts/min, and very hard ≥ 9499 counts/min.
iii. Inter-daily stability (IS): the repetitiveness of the rhythm across consecutive days (i.e., synchronization of the 24 h activity–rest rhythm to the 24 h light–dark cycle). IS ranges from 0 to 1. A high value indicates good synchronization to light and other environmental cues that regulate the biological clock.
iv. Intra-daily variability (IV): fragmentation estimate of the 24 h resting activity rhythm (IV ≈ 0 for a perfect sine wave, IV ≈ 2 for Gaussian noise). A healthy adult has an IV of less than 1.
v. Relative amplitude (RA): the difference between M10 and L5 in the 24 h period. High RA indicates a more robust 24 h rest–activity rhythm. Values near 0 indicate null contrast between wakefulness and sleep, whereas values near 1 express maximal contrast.
vi. Circadian function index (CFI) characterizes the robustness of the rhythm, ranging from 0 to 1.

Complimentary measures

i. Actigraphy also allows the assessment of the chronotype, classifying the participants as early risers versus night owls^7^. Chronotype was quantified by calculating the midpoint between the start and end of SLP^8,9,10^ and midpoint of TIB^11^.
ii. The exposure to sunlight and blue light during the 24 h periods was also quantified. For this, the infrared light record was used to indirectly infer the sunlight, because infrared radiation is 30% to 54% of solar energy^12^. A threshold of 200 lux was used because this is the average illuminance of indoor lighting (at home), and exposure to greater light implies time spent outdoors^13^.

The following scale was used to quantify the levels of blue light intensity: (i) moderate ≥16 lux, (ii) low ≤8 lux, (iii) few <5 lux, and (iv) dark ≤0.01 lux^14,15^.

**Table S1:** Variables that contributed to the formation of clusters.

Variable	Cluster 1 (n = 6)	Cluster 2 (n = 13)	F *(p)*
Time in bed (TIB)	6.76	8.25	24.80 (<0.001)
Sleep period (SLP)	5.67	7.30	33.15 (<0.001)
Sleep duration during TIB (SMIN)	333.55	434.51	40.29 (<0.001)
Sleep duration during SLP (TSMIN)	304.12	408.53	44.93 (<0.001)
Percent sleep (PSLP)	75.10	82.61	8.29 (0.010)

F = ANOVA, p < 0.05; gl= degrees of freedom (1 – 17).

**Table S2:** Differences between weekdays and weekends by clusters.

Sleep parameters	Normal sleepers (n = 13)			Short sleepers (n = 6)		
	Me ± SD	Md (25^th^ – 75^th^)	U (p)^†^	Me ± SD	Md (25^th^ – 75^th^)	U (p)^†^
Bedtime (hh:mm)						
Weekdays	03:36 ± 10.63	02:40 (00:52 – 23:00)	76.00 (0.663)	03:31 ± 7.40	02:20 (01:45 – 03:55)	16.00 (0.749)
Weekend	02:34 ± 10.68	02:40 (00:44 – 23:12)		04:00 ± 5.98	03:30 (01:45 – 03:18)	
Get up time (hh:mm)						
Weekdays	08:58 ± 2.03	09:00 (07:37 – 09:50)	83.00 (0.939)	08:55 ± 1.82	08:01 (07:21 – 10:30)	13.00 (0.423)
Weekend	08:58 ± 1.48	09:00 (07:34 – 09:49)		09:43 ± 2.55	09:27 (07:34 – 10:11)	
TIB (h)						
Weekdays	8.21 ± 0.95	8.16 (7.42 – 8.53)	68.50 (0.418)	7.02 ± 1.18	7.16 (5.55 – 7.54)	7.00 (0.078)
Weekend	7.57 ± 1.24	7.40 (7.29 – 8.55)		6.09 ± 1.47	6.10 (5.10 – 7.00)	
SLP (h)						
Weekdays	7.22 ± 0.98	7.25 (6.46 – 7.52)	76.00 (0.663)	5.55 ± 1.22	5.45 (4.56 – 6.55)	5.00 (0.037)^a^
Weekend	7.07 ± 1.25	6.58 (6.14 – 8.04)		5.03 ± 1.69	5.22 (3.31 – 6.25)	
Onset SLP (hh:mm)						
Weekdays	02:58 ± 9.40	02:12 (02:12 – 02:45)	72.00 (0.522)	03:34 ± 4.06	02:53 (02:06 – 04:21)	9.00 (0.150)
Weekend	03:04 ± 9.51	02:49 (00:50 – 02:20)		03:30 ± 5.81	05:02 (03:00 – 06:00)	
Offset SLP (hh:mm)						
Weekdays	08:44 ± 2.09	08:45 (08:45 – 09:36)	84.00 (0.980)	08:41 ± 2.05	08:00 (07:10 – 10:11)	12.00 (0.337)
Weekend	08:46 ± 1.47	08:43 (07:24 – 09:40)		09:34 ± 2.58	09:12 (07:25 – 10:00)	
Chronotype Midpoint of the TIB (hh:mm)						
Weekdays	05:09 ± 1.58	04:44 (04:00 – 05:42)	84.00 (0.980)	05:31 ± 1.42	05:02 (04:06 – 06:38)	11.00 (0.262)
Weekend	05:09 ± 1.29	04:49 (03:54 – 05:35)		06:44 ± 2.46	05:55 (04:56 – 06:22)	
Midpoint of the SLP (hh:mm)						
Weekdays	05:13 ± 1.77	04:55 (03:51 – 05:48)	83.00 (0.939)	05:45 ± 1.69	05:27 (04:25 – 07:09)	11.00 (0.262)
Weekend	05:14 ± 1.37	04:58 (03:59 – 05:58)		07:05 ± 2.47	06:48 (05:10 – 07:41)	

Data are presented as mean and standard deviation (Me ± SD) and median and quartile [Md [(25th – 75th)]; TIB = Time in bed; SLP = sleep period; NA = number of awakenings; A1′ = awakenings lasting only 1 min; ^†^U(p) = the Mann–Whitney test value for differences between weekdays and weekends; ^a^Significantly different (p < 0.05) between groups in the Mann–Whitney test.

**Table S3:** Light level.

	Normal sleepers (n = 13)		Short sleepers (n = 6)		
Time	Me ± SD	Md (25^th^ – 75^th^)	Me ± SD	Md (25^th^ – 75^th^)	U *(p)*
Daylight (lux)					37.00 (0.015)a
06:00	0.21 ± 0.11	0.24 (0.10 – 0.31)	0.57 ± 0.94	0.14 (0.05 – 1.06)	
07:00	31.20 ± 26.85	27.21 (7.99 – 54.82)	7.56 ± 7.90	6.48 (0.79 – 12.21)	
08:00	76.83 ± 56.79	50.19 (33.87 – 134.40)	16.67 ± 18.23	10.03 (6.67 – 18.60)	
09:00	228.69 ± 95.36	261.75 (123.13 – 314.10)	10.33 ± 5.03	10.44 (6.06 –11.60)	
10:00	318.42 ± 146.50	279.14 (202.08 – 494.74)	41.50 ± 47.40	21.76 (18.75 – 49.55)	
11:00	362.22 ± 156.97	390.58 (195.86 – 517.60)	67.62 ± 105.83	25.34 (17.72 – 56.00)	
12:00	283.21 ± 200.83	185.83 (171.34 – 392.65)	73.68 ± 83.96	48.14 (39.07 – 59.29)	
13:00	307.15 ± 120.35	250.81 (222.22 – 441.29)	102.72 ± 119.18	48.79 (25.35 –260.25)	
14:00	188.11 ± 121.97	181.95 (80.00 – 226.52)	36.74 ± 48.94	16.77 (10.64 – 40.24)	
15:00	170.25 ± 108.45	153.54 (76.67 – 308.58)	22.53 ± 14.23	21.57 (11.34 – 23.91)	
16:00	153.07 ± 49.66	153.37 (110.06 – 177.36)	40.78 ± 26.80	43.90 (12.25 – 62.24)	
17:00	25.02 ± 12.69	21.98 (14.61 – 30.69)	9.06 ± 8.60	6.66 (2.50 – 18.58)	
18:00	1.77 ± 0.66	1.57 (1.44 – 2.29)	0.45 ± 0.12	0.51 (0.29 – 0.53)	
Blue light (lux)					61.00 (0.525)
18:00	3.67 ± 2.94	2.45 (2.04 – 3.75)	1.86 ± 1.05	1.45 (1.21 – 2.14)	
19:00	2.91 ± 1.07	3.19 (1.95 – 3.37)	1.54 ± 0.93	1.27 (0.97 – 1.99)	
20:00	2.78 ± 0.81	2.45 (2.19 – 3.22)	1.44 ± 0.55	1.21 (1.05 – 2.09)	
21:00	2.59 ± 0.45	2.73 (2.12 – 2.88)	1.26 ± 0.27	1.31 (1.10 – 1.52)	
22:00	1.67 ± 0.47	1.80 (1.25 – 2.01)	1.36 ± 0.39	1.17 (1.08 – 1.92)	
23:00	1.16 ± 0.26	1.11 (0.93 – 1.32)	1.00 ± 0.34	0.99 (0.71 – 1.18)	
00:00	0.87 ± 0.20	0.94 (0.66 – 1.01)	0.79 ± 0.27	0.89 (0.67 – 1.01)	
01:00	0.54 ± 0.21	0.59 (0.33 – 0.71)	0.51 ± 0.28	0.43 (0.22 – 0.76)	
02:00	0.20 ± 0.12	0.16 (0.10 – 0.32)	0.23 ± 0.21	0.21 (0.01 – 0.36)	
03:00	0.04 ± 0.04	0.02 (0.01 – 0.06)	0.10 ± 0.12	0.02 (0.01 – 0.17)	
04:00	0.04 ± 0.04	0.02 (0.01 – 0.06)	0.06 ± 0.09	0.02 (0.01 – 0.07)	
05:00	0.22 ± 0.22	0.11 (0.02 – 0.49)	0.13 ± 0.13	0.05 (0.02 – 0.25)	

Data are presented as mean and standard deviation (Me ± SD) and median and quartile [Md [(25th – 75th)]; ^a^*p* < 0.05 in the Mann–Whitney test (U).

**Table S4:** Correlations of light exposure with sleep parameters.

Variables	Correlation
Daylight exposure with sleep parameters	τ (*p*)
Sunlight × onset L5-PIM	−0.32 (0.054)
Sunlight × Onset SLP	−0.07 (0.649)
Blue light exposure with sleep parameters	
Blue light × onset L5-PIM	0.01 (0.972)
Blue light × onset SLP	−0.17 (0.310)
